# The Challenge for Reconstructive Surgeons in the Twenty-First Century: Manufacturing Tissue-Engineered Solutions

**DOI:** 10.3389/fsurg.2015.00052

**Published:** 2015-10-16

**Authors:** Zita M. Jessop, Sarah Al-Himdani, Marc Clement, Iain Stuart Whitaker

**Affiliations:** ^1^Reconstructive Surgery and Regenerative Medicine Research Group (ReconRegen), Institute of Life Science, Swansea University Medical School, Swansea, UK; ^2^The Welsh Centre for Burns and Plastic Surgery, Morriston Hospital, Swansea, UK; ^3^School of Management, Swansea University, Swansea, UK; ^4^Institute of Life Science, Swansea University Medical School, Swansea, UK

**Keywords:** tissue engineering, regenerative medicine, plastic and reconstructive surgery, translational research, biomanufacturing, barriers to translation

## Background

**Figure 1:**
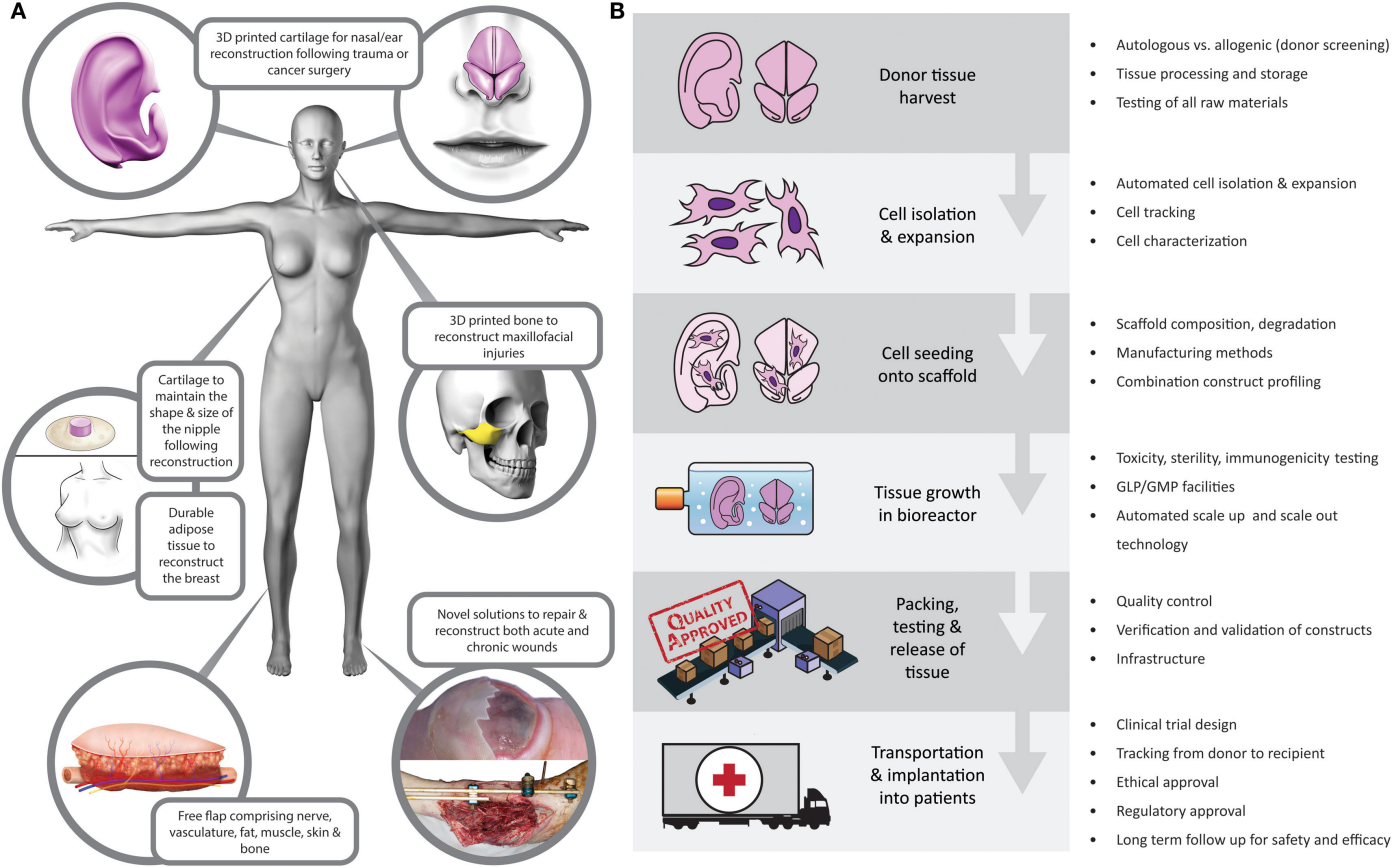
**(A)** Tissue-engineered reconstructive solutions. **(B)** Potential barriers to translation at each level of the tissue-engineering supply chain.

These are exciting times in the field of plastic and reconstructive surgery. Meaningful advances in a wide range of basic science and clinical spheres have been made in the field in recent years, with direct translation to patient care. As a community, we are fortunate to be involved in such a vast, complex, increasingly interdisciplinary, rapidly expanding, and intellectually challenging field of surgery. Plastic surgery aims to restore “form and function” following a wide range of congenital or acquired defects, with procedures often transcending anatomic boundaries. This versatility promotes innovation, and with the recent advances in medical imaging (1), microsurgery (2), composite tissue allotransplantation (3, 4), nanotechnology (5), cell biology and biomaterials (6), and 3D printing (7), treatment options for patients are wider than ever before. For centuries, the “reconstructive ladder” was restricted to local flaps and skin grafts. Although these autologous options are reliable, plastic surgeons, with their constant wish to refine techniques, have become increasingly cognizant that there is the real potential for a paradigm shift in reconstructive surgery in the medium term. Tissue-engineered solutions (Figure 1A) offer the potential to alleviate the need for donor sites and their associated morbidity and to reduce hospital stay and associated costs (8).

## Extent of the Problem

Reconstructive plastic surgeons have a diverse workload and contrary to the perceptions of the media, it comprises a relatively small proportion of purely aesthetic procedures (9). The majority of operations relate to wound management and neoplasia, with a significant health economic impact (9). In the year 2013–2014 alone, over one million patients were treated in NHS England by plastic surgeons (10, 11). Evidence suggests that this workload will continue to increase (12). With a continual supply of major trauma and neoplasia, plastic surgeons are facing more challenging composite defects than ever before coupled with internet and media savvy patients with increasing expectations (13).

## Innovations in Reconstructive Surgery

Technological innovation in plastic and reconstructive surgery in the twentieth century revolutionized the specialty, opening up the possibility for surgeons to operate on microvascular structures using specialized microscopes and instruments, enabling them to undertake free tissue transfers (2) and extremity replantations. Despite these developments in practice, we are still confronted with shortcomings relating to the availability of donor tissues. In order to overcome this, novel approaches have been investigated. Transplantation of organs and tissues, first performed by a plastic surgeon and Nobel laureate (Dr. Joseph Murray) in 1954 (14, 15), has rapidly expanded over the twentieth century from traditional solid organ transplant to a range of vascularized composite tissue allografts including the upper extremity (3) and face (4). Although excellent medium-term outcomes have been reported, there have been ongoing debates surrounding ethical (16, 17), psychological, and medical issues in the long term, especially relating to the effects of immunosuppression (18). The more attractive concept, obviating the need for long-term immunosuppression, is tissue engineering. The Medical Research Council states that regenerative medicine and tissue engineering “holds the promise of revolutionizing patient care in the twenty-first century” and the UK government highlighted regenerative medicine as one of the key areas, which could provide a global competitive advantage for the UK (19, 20). In 2014, it was highlighted as one of the eight great technologies in the industrial strategy government manifesto worthy of significant investment.

## The Challenges

Tissue engineering is a modern, interdisciplinary field combining principles of engineering, physics, and life sciences. It shares a common objective with plastic and reconstructive surgery: “to restore form and function” (21, 22). The surgical community worldwide is becoming increasingly aware of the research landscape. The American Society of Plastic surgeons have highlighted the role of tissue engineering in the future of plastic surgery (23), particularly the need for a focus on translation from bench research to clinical practice, which would provide clarity on avenues for upscaling and tissue engineering supply chains (Figure 1B).

Although the UK government and others have recognized the potential of regenerative medicine and tissue engineering to impact on the health service, they also highlighted the current “lack of coordination” in the field as a whole (24). Despite recent *in vitro* and *in vivo* studies attesting to the feasibility of tissue engineering for use in a range of clinical scenarios (25–30), barriers have been identified that are inhibiting clinical translation (Figure 1B). These barriers to translation are deep rooted at the basic science level, manifesting in insufficient understanding of the safety and durability of tissue-engineered constructs (31, 32). Understandably, before these hurdles are overcome, investors are unwilling to shoulder the financial, ethical, and regulatory burden of translational research that may not be commercially viable, which then impacts on funding and further progress in the field (33).

### Scientific Unknowns

The term “tissue engineering” was first coined almost 30 years ago, with skin being one of the first to be used clinically (34). Despite numerous successes in this exciting field, there have been very few reports of successful clinical translation. This is due to a combination of factors, including lack of clarity regarding ideal cell sources and scaffolds (35), methods of vascularizing larger constructs (36), reliable cell and construct characterization preimplantation and evidence of safety (37), and durability in large animal models (35). This is not surprising given the complexity of research in this field that cross cuts cell biology, material science, biochemistry, and engineering. As cells are complex, dynamic, and interactive, it is crucial to have a solid scientific foundation regarding their stability, functional capacity, and response to host environment (38). It is generally accepted that it takes 20–30 years from the start of basic science research to clinical utility, demonstrated by contemporary treatments such as bone marrow transplantation (39).

### Ethical and Regulatory Milestones

There is a lack of international standards for the use of stem cell and tissue engineering therapies in human patients, which has led to the use of unproven treatments in unregulated international clinics which damages the research in this field (40, 41). Stem cell hype has also generated unrealistic patient hopes, which raises ethical concerns when initiating clinical trials of cell-based therapies (42). Tissue-engineered constructs do not easily fit into the U.S. Food and Drug Administration (FDA) and the European Medicines Agency (EMA) classifications for medical products (43). Since many of these constructs are complex and consist of more than one component, i.e., biomolecule, cell, and biomaterial, they are classed as combination products that fall under the advanced therapy medicinal products (ATMPs) category (44, 45). The review of any regulated product is therefore undertaken on a case-by-case basis but generally considers four aspects: product manufacture, preclinical testing (laboratory and animal models), clinical performance, and product labeling according to claim of intended use. These processes are time, labor, and money intensive and have often been criticized for lack of clarity and inconsistencies between different regulatory agencies (46, 47), which have in turn hindered the availability of products in some areas. The tissue-engineering field is still in early development and hence regulatory requirements are also continuing to evolve with it. There is now increasing dialog between the international TERMIS committees and regulatory bodies in order to work toward a consensus on regulatory requirements in order to foster innovation while continuing to ensure the safety and efficacy of tissue-engineered solutions (46).

### Lack of Funding

Despite investment in the tissue-engineering sector from a number of companies, the high production costs, unrealistic promises from the scientific community, and regulatory hurdles have limited successful clinical translation and commercial success of these early products (48–50). It has been suggested that alliances between companies or collaboration between industry and government funding agencies, who can share expertise as well as the financial burden, will be the key to driving translation from bench to bedside through clinical trials (33, 49). Partnerships within and across different sectors, such as the CASMI Translational Stem Cell Consortium (CTSCC) (51) (an academic-industry consortium), are already emerging and aim to identify key hurdles in commercialization. There is also an increasing focus on developing “translation centers” such as Harvard Stem Cell Institute (HSCI), Centre for Commercialisation of Regenerative Medicine (CCRM), and the Cell Therapy Catapult (51).

### Infrastructure for Scale Up and Scale Out

Biomanufacturing is an often overlooked but vital process of clinical translation (52). Once a successful tissue-engineered construct has been developed and tested in the laboratory, there are a number of considerations when driving the product toward clinical trials and widespread commercial use. Many methods of cell isolation, expansion, differentiation, and seeding onto scaffolds continue to be variable bench processes. These processes need to be refined by using batch-tested reagents, defined culture and bioreactor conditions, and automated techniques to ensure the process is quality controlled and able to be verified and validated according to regulatory requirements (38). A key component is the transition from manual laboratory based techniques to certified good manufacturing practice (cGMP) processes, which require a sound business model, significant infrastructure, personnel and automated scale-up and scale-out strategies. In order to set up these processes for widespread use in health services, there needs to be synergized communication between basic researchers, clinicians, industry experts, and regulators. There are initiatives to set up translational hubs for ATMPs, such as the European Advanced Translational Research Infrastructure in Medicine (EATRIS), in order to fill the gap between basic science and clinical practice and overcome the bottlenecks for translation (45).

## Conclusion

There is a clear and unmet clinical need for an adequate solution to reconstruct tissue defects following tumor resections, severe trauma, or deep burns, which removes the morbidity and mortality associated with donor sites. Tissue engineering, with its potential to biomanufacture constructs to repair and restore complex defects, holds great promise (53). The innovative and complex nature of these constructs results in a number of scientific, ethical, regulatory, financial, and infrastructural challenges, which currently hinder clinical translation. Answers to the fundamental scientific questions as well as our ability to overcome the translational challenges rely on multidisciplinary working groups (academia, clinicians, patient representatives, investors, manufacturing experts, and regulators) as well as governmental support (54) before we will see widespread use of tissue-engineered constructs in reconstructive surgery.

## Author Contributions

ZJ and SA-H undertook the literature review and drafted the manuscript. MC revised the manuscript. IW conceived the study and revised the manuscript. All authors read and approved the final manuscript.

## Conflict of Interest Statement

The authors declare that the research was conducted in the absence of any commercial or financial relationships that could be construed as a potential conflict of interest.

## References

[1] Pratt GF Rozen WM Chubb D Ashton MW Alonso-Burgos A Whitaker IS. Preoperative imaging for perforator flaps in reconstructive surgery: a systematic review of the evidence for current techniques. Ann Plast Surg (2012) 69:3–9. 10.1097/SPA.0b013e318222b7b7 22627495

[2] Taylor GI Daniel RK The free flap: composite tissue transfer by vascular anastomosis. Aust N Z J Surg (1973) 43:1–3. 10.1111/j.1445-2197.1973.tb05659.x 4200573

[3] Shores JT Brandacher G Lee WP. Hand and upper extremity transplantation: an update of outcomes in the worldwide experience. Plast Reconstr Surg (2015) 135:351e–60e. 10.1097/PRS.0000000000000892 25401735

[4] Khalifian S Brazio PS Mohan R Shaffer C Brandacher G Barth RN Facial transplantation: the first 9 years. Lancet (2014) 384:2153–63. 10.1016/S0140-6736(13)62632-X 24783986

[5] Nodzo SR Hohman DW Chakravarthy K Nanotechnology: why should we care? Am J Orthop (2015) 44:E87–8. 25750958

[6] Naderi H Matin MM Bahrami AR. Review paper: critical issues in tissue engineering: biomaterials, cell sources, angiogenesis, and drug delivery systems. J Biomater Appl (2011) 26:383–417. 10.1177/0885328211408946 21926148

[7] Murphy SV Atala A 3D bioprinting of tissues and organs. Nat Biotechnol (2014) 32:773–85. 10.1038/nbt.2958 25093879

[8] Golas AR Hernandez KA Spector JA. Tissue engineering for plastic surgeons: a primer. Aesthetic Plast Surg (2014) 38:207–21. 10.1007/s00266-013-0255-5 24378377

[9] Mason L Whitaker L Boyce D Dismissing the myths: an analysis of 12,483 procedures. All in a years work for a plastic surgical unit. The Internet Journal of World Health and Societal Politics (2008) 6(2).

[10] Hospital Episode Statistics, Admitted Patient Care, England 2012–13. Health and Social Care Information Centre [Cited 2015 Oct 7]. Available from: http://www.hscic.gov.uk/catalogue/PUB12566

[11] Hospital Episode Statistics, Outpatient care, April 2015. Health and Social Care Information Centre [Cited 2015 Oct 7]. Available from: http://www.hscic.gov.uk/searchcatalogue?productid=18356&q=title%3a%22hospital+episode+statistics%22&sort=Relevance&size=10&page=1#top

[12] Recommendation for Plastic Surgery Training 2011 UK: Centre for Workforce Intelligence a Report from the Royal College of Surgeons of England [Cited 2015 Oct 7]. Available from: http://www.rcseng.ac.uk/surgeons/surgical-standards/docs/2011-surgical-workforce-census-report

[13] Søreide K (2009). Epidemiology of major trauma. Br J Surg 96:697–8. 10.1002/bjs.6643 19526611

[14] Harrison JH Merrill JP Murray JE Renal homotransplantation in identical twins. Surg Forum (1956) 6:432–6. 13391513

[15] Cannon B Murray JE Plastic surgery: tissue and organ homotransplantation. N Engl J Med (1956) 255:900–4. 10.1056/NEJM195611082551906 13369737

[16] Wiggins OP Barker JH Martinez S Vossen M Maldonado C Grossi F On the ethics of facial transplantation research. Am J Bioeth (2004) 4(3):1–12. 16192123 10.1080/15265160490496507

[17] McDowell N Surgeons struggle with ethical nightmare of face transplants. Nature (2002) 420:449 10.1038/420449a 12466799

[18] Whitaker IS Duggan EM Alloway RR Brown C McGuire S Woodle ES Composite tissue allotransplantation: a review of relevant immunological issues for plastic surgeons. J Plast Reconstr Aesthet Surg (2008) 61:481–92. 10.1016/j.bjps.2007.11.019 18248779

[19] Committee GBP HOLSAT. House of Lords – Select Committee on Science and Technology – HL 76. London: The Stationery Office Limited (2013).

[20] O’Dowd A Peers call for UK to harness “enormous” potential of regenerative medicine. BMJ (2013) 347:f4248 10.1136/bmj.f4248 23818565

[21] Sterodimas A De Faria J Correa WE Pitanguy I. Tissue engineering in plastic surgery: an up-to-date review of the current literature. Ann Plast Surg (2009) 62:97–103. 10.1097/SAP.0b013e3181788ec9 19131730

[22] Skalak R Fox CF Tissue Engineering. Alan R. Liss, New York (1988).

[23] D’mico RA Rubin JP Regenerative medicine and the future of plastic surgery. Plast Reconstr Surg (2014) 133:1511–2. 10.1097/PRS.0000000000000212 24867733

[24] House of Lords Science and Technology Committee. 28 Jun 2013. [Cited 2015 Oct 7]. Available from: http://www.parliament.uk/hlscience

[25] Kim YS Choi YJ Lee SW Kwon OR Suh DS Heo DB Assessment of clinical and MRI outcomes after mesenchymal stem cell implantation in patients with knee osteoarthritis: a prospective study. Osteoarthritis Cartilage (2015):S1063–4584. 10.1016/j.joca.2015.08.009 26318655

[26] Findlay MW Dolderer JH Trost N Craft RO Cao Y Cooper-White J Tissue-engineered breast reconstruction: bridging the gap toward large-volume tissue engineering in humans. Plast Reconstr Surg (2011) 128:1206–15. 10.1097/PRS.0b013e318230c5b2 22094739

[27] Georgiou M Golding JP Loughlin AJ Kingham PJ Phillips JB. Engineered neural tissue with aligned, differentiated adipose-derived stem cells promotes peripheral nerve regeneration across a critical sized defect in rat sciatic nerve. Biomaterials (2015) 37:242–51. 10.1016/j.biomaterials.2014.10.009 25453954

[28] Patrick CW. Breast tissue engineering. Annu Rev Biomed Eng (2004) 6:109–30. 10.1146/annurev.bioeng.6.040803.140032 15255764

[29] Bichara DA O’Sullivan N-A Pomerantseva I Zhao X Sundback CA Vacanti JP The tissue-engineered auricle: past, present, and future. Tissue Eng Part B Rev (2012) 18:51–61. 10.1089/ten.TEB.2011.0326 21827281

[30] Reichert JC Hutmacher DW Bone tissue engineering. In: Pallua N Suscheck CV, editors. Tissue Engineering. Berlin, Heidelberg: Springer (2010). p. 431–56.

[31] Gad SC McCord MG Safety Evaluation in the Development of Medical Devices and Combination Products. 3rd ed Cary: CRC Press (2008).

[32] Derksen M-H Engineering Flesh; Towards Professional Responsibility for “Lived Bodies” in Tissue Engineering. Eindhoven: 3TU Ethics (2008).

[33] Hewson SM Fehlings LN Messih M Fehlings MG. The challenges of translating stem cells for spinal cord injury and related disorders: what are the barriers and opportunities? Expert Rev Neurother (2013) 13:143–50. 10.1586/ern.12.157 23368801

[34] Keck M Lumenta DB Kamolz L-P Skin tissue engineering. In: Kamolz LP Lumenta DP, editors. Dermal Replacements in General, Burn, and Plastic Surgery. Vienna: Springer (2013). p. 13–25.

[35] Ikada Y Challenges in tissue engineering. J R Soc Interface (2006) 3:589–601. 10.1098/rsif.2006.0124 16971328 PMC1664655

[36] Novosel EC Kleinhans C Kluger PJ. Vascularization is the key challenge in tissue engineering. Adv Drug Deliv Rev (2011) 63:300–11. 10.1016/j.addr.2011.03.004 21396416

[37] Klinger RY Blum JL Hearn B Lebow B Niklason LE. Relevance and safety of telomerase for human tissue engineering. Proc Natl Acad Sci U S A (2006) 103:2500–5. 10.1073/pnas.0508184103 16477025 PMC1413782

[38] Parenteau NL. Commercial development of cell-based therapeutics: strategic considerations along the drug to tissue spectrum. Regen Med (2009) 4:601–11. 10.2217/rme.09.29 19580408

[39] Nakano T Era T Kodama H Honjo T Development of blood cells from mouse embryonic stem cells in culture. In: Ikehara S Takaku F Good RA, editors. Bone Marrow Transplantation. Tokyo: Springer Science & Business Media (2012). p. 9–19.

[40] Cohen CB Cohen PJ International stem cell tourism and the need for effective regulation. Part I: Stem cell tourism in Russia and India: clinical research, innovative treatment, or unproven hype? Kennedy Inst Ethics J (2010) 20:27–49. 10.1353/ken.0.0305 20506693

[41] Lau D Ogbogu U Taylor B Stafinski T Menon D Caulfield T. Stem cell clinics online: the direct-to-consumer portrayal of stem cell medicine. Cell Stem Cell (2008) 3:591–4. 10.1016/j.stem.2008.11.001 19041775

[42] The darker side of stem cells. Nature (2012) 483:5 10.1038/483005a 22382939

[43] Panetta NJ Gupta DM Longaker MT Bench to bedside: navigating industry, the FDA and Venture Capital. In: Chang J Gupta G editors. Tissue Engineering for the Hand. World Scientific Publishing Co. (2010). p. 253–69.

[44] Hellman KB Engineered tissues: the regulatory path from concept to market. In: Fisher JP, editor. Tissue Engineering Advances in Experimental Medicine and Biology. Boston, MA: Springer p. 363–76. 10.1007/978-0-387-34133-0_23 17120795

[45] Belardelli F Rizza P Moretti F Carella C Galli MC Migliaccio G. Translational research on advanced therapies. Ann Ist Super Sanita (2011) 47:72–8. 10.4415/ANN_11_01_15 21430343

[46] Bertram T Hellman KB Bayon Y Ellison S Wilburn SRS The regulatory imperative: international perspective. Tissue Eng Part B Rev (2013) 19:191–3. 10.1089/ten.teb.2012.0654 23216282

[47] Bertram TA Tentoff E Johnson PC Tawil B Van Dyke M Hellman KB. Hurdles in tissue engineering/regenerative medicine product commercialization: a pilot survey of governmental funding agencies and the financial industry. Tissue Eng Part A (2012) 18:2187–94. 10.1089/ten.TEA.2012.0186 22838399

[48] Wilan KH Scott CT Herrera S Chasing a cellular fountain of youth. Nat Biotechnol (2005) 23:807–15. 10.1038/nbt1005-1315c 16003362

[49] Pangarkar N Hutmacher DW. Invention and business performance in the tissue-engineering industry. Tissue Eng (2004) 9:1313–22. 10.1089/10763270360728224 14670118

[50] Trounson A Integration and translation: driving stem cell therapies toward the clinic. Regen Med (2008) 3:269–73. 10.2217/17460751.3.3.269 18462051

[51] French A Bure K Brindley DA. CASMI TSCC launch event, Paris, France, July 2013: an assessment of the key barriers to the commercialization and clinical adoption of pluripotent stem cell therapies. Rejuvenation Res (2014) 17(1):84–8. 10.1089/rej.2014.1545 24392658 PMC3929322

[52] French A Buckler RL Brindley DA. Commercialization of regenerative medicine: learning from spin-outs. Rejuvenation Res (2013) 16(2):164–70. 10.1089/rej.2013.1423 23470045

[53] Pallua N Suschek CV Tissue Engineering: From Lab to Clinic. Berlin, Heidelberg: Springer (2010).

[54] Pallua N Gröger A Tissue engineering and plastic surgery. In: Eisenmann-Klein M Nuehann-Lorenz C, editors. Innovations in Plastic and Aesthetic Surgery. Berlin, Heidelberg: Springer (2010). p. 17–23.

